# Strangulation of the Penis by a Metallic Ring: Prevention Is Better Than Cure

**DOI:** 10.1155/2018/1725752

**Published:** 2018-03-13

**Authors:** Hamza Ichaoui, Sataa Sallami, Ahmed Samet, Zied Bokal, Hassan Touinsi

**Affiliations:** Department of Surgery, Mohamed Tahar Maamouri Hospital, Nabeul, Tunisia

## Abstract

Strangulation of the penis is a rare condition that requires emergency management. Several objects, metallic or not, can be placed on the penis to increase sexual performance or for self-erotic intentions especially in psychotic patients with unusual sexual impulses. The problem of removing the foreign body and repairing the damage is a real challenge for the practitioner and a great stress for the patient. We report a case of a 42-year-old schizophrenic patient who presented to the emergency department for a strangulation of the penis secondary to a metal ring placed at the base of the penis 10 days before the consultation without urinary disorder. A review of the literature allowed us to highlight the different clinical pictures of penis strangulation and the therapeutic methods and to highlight the importance of psychiatric care of sexual behaviour in chronic psychotics.

## 1. Introduction

Schizophrenic patients frequently have very poor interpersonal relationships. Disability comes from the association of different psychic symptoms that disrupt interactions with others and inevitably influence their sexual life. The erroneous perception of the external genital organs, decreased libido, and erectile dysfunction due mainly to neuroleptics can lead these subjects to unusual sexual practices such as self-mutilations or, as in our case, putting a ring at the base of the penis.

We report a case of strangulation of the penis by a metallic ring. The therapeutic circumstances and approach are given in detail in this work.

## 2. Note

A man, aged 42, who is single, schizophrenic, and under neuroleptic and anticholinergic therapy (chlorpromazine, haloperidol, and trihexyphenidyl), presented to the emergency department with his brother to consult for strangulation of the penis by a metallic ring placed at the base of the penis 10 days before for self-erotic stimulation.

The clinical examination found a bluish edema with blisters and ulcers on both sides. The glans was engorged and cyanosed, with a total loss of sensitivity. There was neither urethral fistula nor urinary disorders associated. The patient was calm without any signs of anxiety.

**Figure 1:**
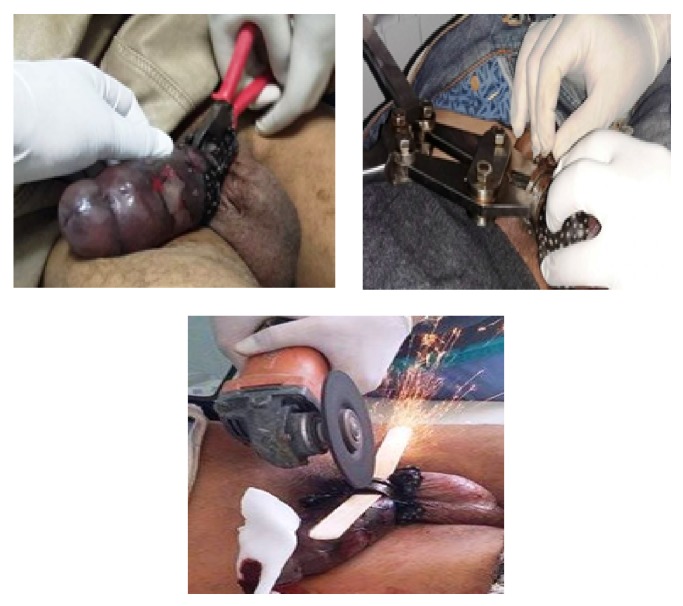
Different materials used for cutting the metal ring incarcerated in the root of the penis.

Attempts to remove the ring by lubrication, clamping, and then a manual saw were unsuccessful. We have made a section of the metallic ring by an angle grinder. A wooden tongue depressor was inserted between the penis and the ring and permanent cooling with physiological serum was performed (Figure 1).

**Figure 2:**
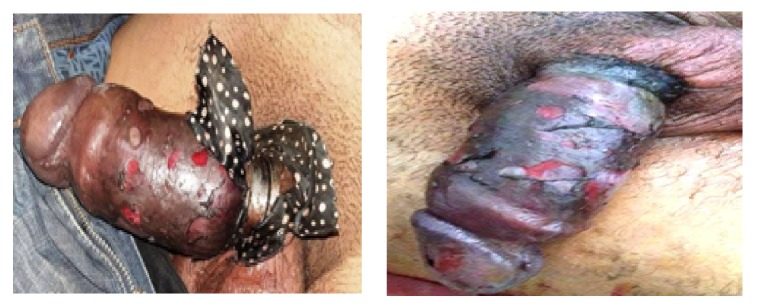
Condition of the penis before and after removing the ring.

The sequences were marked by suppuration of the cutaneous lesions (24 hours after removal of the metal ring) (Figure 2).

The diagnosis was made of wet gangrene and the patient was given piperacillin, metronidazole, and amikacin with local care.

After the end of the antibiotic treatment and drying of the lesions, the patient was addressed to psychiatry. The penis was swollen, blackish, and insensible. The patient was not seen again.

## 3. Discussion

Incarceration of genital organs is a rare clinical fact, which poses little diagnostic problem. However, the objective is to find the best method for the ablation of the metal and to appreciate the importance of the damage caused. It is a rare global phenomenon and a circumstantial pathology that most urologists encounter not more than once in their practice.

According to many authors, the incarceration of genital organs in adults is the result of self-erotic acts whose goal is to prolong the erection and delay ejaculation and sometimes to increase manhood (supernatural effect) [1, 2]. In addition, disinhibition or unusual sexual urges may occur in some people with acute symptoms of schizophrenia or other psychosis. These people are, therefore, more likely to have bizarre and dangerous sexual behaviors, such as the case of our patient who believed to have eternal pleasure in incarcerating a metal ring in the penis. In the pediatric population, this maneuver has been attempted to prevent enuresis [3]. The most frequent questioned objects, besides the metal also found in our observation, are hair, threads, fabrics, plastics, laces, and ropes [2, 4]. These objects are responsible for the interruption of the venous and lymphatic return, causing the edema which appears after a few hours and hinders the extraction of the ring. If the compression persists, the arterial flow may be compromised [1, 2]. Several possible complications of strangulation can occur: urinary retention in 50% of cases, urethral fistulas, priapism, distal hypoesthesia, cutaneous ulceration, and necrosis, which can evolve towards gangrene or amputation of the penis. These complications depend on the duration and severity of the compression [1, 5–8].

The principle of the treatment is simple: it is the rapid decompression of the penis to allow a good vascularization of the tissues. On the other hand, the choice of the therapeutic method is a real challenge for the urologist given the great number of ring types used and the variability of the lesions [1].

To facilitate the therapeutic decision, Bhat et al. have developed a simple classification of trauma by strangulation of the penis [5].

*Grade I*. Isolated distal edema.

*Grade II*. Urethral and cutaneous trauma, compression of the corpus spongiosum, and distal hypoesthesia.

*Grade III*. Urethral and cutaneous trauma with loss of distal sensitivity.

*Grade IV*. Rupture of the corpus spongiosum and/or urethral fistula, compression of the corpus cavernosum, and distal anesthesia.

*Grade V*. Gangrene, necrosis, or distal amputation of the penis.

Several therapeutic varieties are proposed to take care of this type of lesions but the choice remains difficult given the unusual nature of this trauma. This choice can be directed by the grade of the lesions [2].

The first step is relief of urinary retention by transurethral catheterisation for grades I and II [9], while cystostomy is preferable for higher grades [10, 11].

The main therapeutic techniques are grouped into 4 categories [2].

*(1) The Thread Method*. It consists of using a silk thread or a latex strip to compress the edematous area, which facilitates the sliding of the ring. It may also be associated with aspiration of the blood by the glans [12]. This technique gives good results for grades I, II, and III and allows decompression without tissue damage [13–15].

*(2) Aspiration*. Needles are used to aspirate the blood of the glans and corpus cavernosum or to make subcutaneous punctures to evacuate the lymph that causes edema [2, 5, 16, 17].

*(3) The Cutting of the Ring*. It depends on the thickness and the materials that make up the ring. This method is mainly used for grades I to III [10]. It requires equipment ranging from a simple hand clamp to a compressed air saw or dental micromotor [18], which are not always available in urology departments [19].

*(4) Decompression Surgery*. It is recommended especially for grades V and consists of a denudation of the penis to the fascia of Buck followed by a graft of skin [8, 20].

After the urgent removal of the obstacle, a psychiatric care is necessary [21]. According to Rizettou and McCann [22, 23], the consideration of sexuality in chronic psychotic patients is often underestimated by therapists, yet it is striking how delusions and hallucinations often have a sexual theme. This lack of care can lead to unusual and often dangerous sexual behaviors such as mutilations and strangulations of the external genital organs, the introduction of foreign bodies into the urethra, the vagina, and the anus, prostitution, rape, and so on. Therefore, sexuality must have its place in psychiatric interviews and psychosocial care must be adapted to each patient in order to prevent this type of accident.

## 4. Conclusion

Strangulation of the penis is a rare trauma that requires management in the first few hours. We think that this type of accident in psychotic patients has a bad prognosis in consequence of the delay of the consultation and the high risk of recurrence for lack of psychiatric care of their sexual disorders. We believe that the prevention of this accident by a more cautious psychiatric care is better than curing such a dangerous trauma.
